# Prevalence of Mental Disorders and Impact on Quality of Life in Patients With Pulmonary Arterial Hypertension

**DOI:** 10.3389/fpsyt.2021.667602

**Published:** 2021-05-31

**Authors:** Karen M. Olsson, Tanja Meltendorf, Jan Fuge, Jan C. Kamp, Da-Hee Park, Manuel J. Richter, Henning Gall, Hossein A. Ghofrani, Pisana Ferrari, Ralf Schmiedel, Hans-Dieter Kulla, Ivo Heitland, Nicole Lepsy, Madelaine-Rachel Dering, Marius M. Hoeper, Kai G. Kahl

**Affiliations:** ^1^Department of Respiratory Medicine, German Center for Lung Research Biomedical Research in Endstage and Obstructive Lung Disease Hannover (DZL/BREATH), Hannover Medical School, Hannover, Germany; ^2^Department of Psychiatry, Social Psychiatry and Psychotherapy, Hannover Medical School, Hannover, Germany; ^3^Department of Internal Medicine, German Center for Lung Research (DZL), Justus Liebig University Giessen, Universities of Giessen and Marburg Lung Center, Giessen, Germany; ^4^Department of Pneumology, German Center for Lung Research (DZL), Kerckhoff Heart, Rheuma and Thoracic Center, Universities of Giessen and Marburg Lung Center, Bad Nauheim, Germany; ^5^Italian Pulmonary Hypertension (PH) Association Associazione Ipertensione Polmonare Italiana (AIPI), Bologna, Italy; ^6^Pulmonale Hypertonie Selbsthilfe, Bottrop, Germany; ^7^Pulmonale Hypertonie eV, Rheinstetten, Germany

**Keywords:** pulmonary arterial hypertension, anxiety disorder, adjustment disorder, psychocardiology, quality of life, depression disorder, panic disorder

## Abstract

**Objective:** Mental health may affect the quality of life (QoL) in patients with pulmonary arterial hypertension (PAH). However, mental disorders have not been systematically assessed in these patients. We examined the prevalence of mental disorders using structured interviews and determined their impact on QoL in patients with PAH.

**Methods:** This study included 217 patients with PAH from two German referral centers. Psychiatric disorders were assessed using the structured clinical interview for DSM-V. QoL was assessed using the WHO Quality of Life questionnaire (short form). The diagnostic value of the Hospital Anxiety and Depression Scale was evaluated by receiver operating characteristic curve analysis.

**Results:** More than one third of the patients had psychological disorders with current or past adjustment disorder (38.2%), current major depressive disorder (23.0%), and panic disorder (15.2%) being the most prevalent mental illnesses. About half of the patients with a history of adjustment disorder developed at least one other mental illness. The presence of mental disorders had a profound impact on QoL. The Hospital Anxiety and Depression Scale ruled out panic disorder and depression disorder with negative predictive values of almost 90%.

**Conclusion:** Mental disorders, in particular adjustment disorder, major depression, and panic disorder, are common in patients with PAH and contribute to impaired QoL in these patients. The Hospital Anxiety and Depression Scale may be used as a screening tool for the most common mental health disorders. Future studies need to address interventional strategies targeting mental disorders in patients with PAH.

## Introduction

Pulmonary arterial hypertension (PAH) is a rare debilitating and life-threatening disease characterized by progressive pulmonary vascular remodeling and consecutive right-sided heart failure (1). Despite therapeutic advances, there is yet no cure for PAH. For the affected patients, experiencing the symptoms of the disease, especially the progressive impairment in exercise capacity, together with the uncertainties accompanying the usual diagnostic delay (2, 3) is often traumatizing. Receiving the diagnosis of PAH is frequently associated with conflicting emotions as patients may be relieved that an explanation for their symptoms has been found while realizing at the same time that they suffer from a severe, permanent, and potentially lethal disease, which will affect and change the rest of their lives. The consequences of receiving a PAH diagnosis are immense as the patients have to deal not only with their physical limitations but also with uncertainties about their future; the potential inability to continue working with all its financial, familial, and social consequences; the outlook of not being able to become pregnant or to raise children; the need for continued medical check-ups including invasive procedures; and the burden, complexities, and side effects of medical therapies including the possible need for lung transplantation.

Hence, it is not surprising that some patients may develop an adjustment disorder, which may evolve into other psychiatric conditions during the course of their illness. Symptoms of depression and anxiety have been reported in up to 50% of patients with PAH (4–12). It is likely that such symptoms have a profound effect on the quality of life (QoL) in the affected patients. However, to the best of our knowledge, all previous studies assessing mental disorders in patients with PAH have used self-rating questionnaires rather than structured expert face-to-face interviews, which are considered the gold standard of psychiatric assessment.

In the present cross-sectional study, we assessed the current prevalence of mental disorders in patients with PAH using structured face-to-face interviews using the structured clinical interview (SCID) for DSM-V (13). The prevalence of adjustment disorders after receiving the PAH diagnosis was assessed retrospectively in most cases. In addition, we analyzed the association between mental disorders and QoL. Finally, we investigated the usefulness of the Hospital Anxiety and Depression Scale (14), an established patient self-assessment tool, as a screening instrument for SCID-derived mental disorders in patients with PAH.

## Methods

### Design and Study Setting

This cross-sectional observational multicentre study included patients with a confirmed diagnosis of PAH (WHO Group 1) according to current diagnosis criteria (1), age ≥18 years, and physical and mental capability to complete questionnaires and interview in German language. The study concept was developed in cooperation with patient organizations as suggested during the latest Pulmonary Hypertension World Symposium (15). Patients were enrolled in two German pulmonary hypertension referral centers (Hannover Medical School and University of Gießen and Marburg). The study was approved by the local institutional review boards (Nr. 8540_BO_K_2019 for Hannover and Nr. 21119 for Gießen and Marburg), and all patients gave written informed consent.

### Recruitment

In Hannover, all active patients with PAH deemed eligible were identified from a database and approached via mail. In Gießen, all patients with ambulatory visits between January 1, 2020 and March 31, 2020 were invited to participate. After providing written informed consent, patients completed a set of questionnaires and underwent a personal structured clinical interview for DSM-V.

### Questionnaires and SCID

Assessment of anthropometric data [age, height, weight, body mass index (BMI)], lifestyle factors such as smoking habits (active, former, current, pack years), and current alcohol consumption (drinks per week) and psychosocial data with information regarding educational and employment status were assessed using self-rated questionnaires.

Psychiatric characterization was performed using the Structured Clinical Interview for DSM-V to determine axis-1 psychiatric disorders and covered the past 4 weeks before the interview. The prevalence of adjustment disorders within 3 months after the PAH diagnosis was assessed retrospectively using the same set of questions given in the structured clinical interview. Further characterization comprised symptoms of panic disorder and depression with the Hospital Anxiety and Depression Scale (14), and QoL with the WHO Quality of Life questionnaire in short form (WHOQOL-BREF) (16). QoL was classified as overall, psychological, and physical QoL.

### Psychiatric Diagnosis

Mental disorders were defined according to the Diagnostic and Statistical Manual of Mental Disorders, Fifth Edition (DSM-5).

### Clinical Assessments

Clinical assessments at the time of the study included BMI, blood gas analyses, lung function tests including diffusion capacity of the lungs for carbon monoxide (DLCO), 6-min walk distance (6MWD), WHO functional class (FC), and serum levels of the N-terminal fragment of proBNP (NT-proBNP). Hemodynamic parameters at diagnosis were documented.

### Comparison With General Population

To compare the prevalence of mental disorders in patients with PAH with the prevalence of mental disorders in the general population, data from a subsample aged 18–70 years of the mental health module of the German Health Interview and Examination Survey for Adults (DEGS1-MH; *n* = 5,381) were used (17).

### Statistical Analysis

IBM SPSS Statistics 27.0 (IBM Corp, Armonk, NY, USA) and STATA 13.0 (StataCorp LP, College Station, Texas, USA) statistical software were used for analysis of the data. Continuous variables are shown as median and interquartile range (IQR) or as mean and SD, as appropriate. Categorical variables are shown as n and percent (%) unless indicated otherwise. To compare groups, χ^2^ test, Mann–Whitney *U* test, or *t*-test was used as appropriate. Prevalence rates of mental disorders in PAH and a representative German population (DEGS1-MH study) were compared using χ^2^ test. Point estimates with 95% CIs and differences between the two populations were calculated. To assess effect sizes, Cohen's d for continuous or risk ratios for categorical variables were calculated. To examine the impact of mental disorders on QoL, linear regression models were calculated to assess determinants on QoL (psychological, physical, and overall QoL were used as dependent variables). Using receiver operating characteristic (ROC) curve analysis with calculation of the area under the curve (AUC), the best cut-off values of Hospital Anxiety and Depression Scale A (for anxiety) and D (for depression) with the corresponding sensitivity and specificity on the structured clinical interview–derived outcomes panic disorder and depression disorder were calculated. *P*-values <0.05 were considered statistically significant.

## Results

### Participant Characteristics

**Figure 1:**
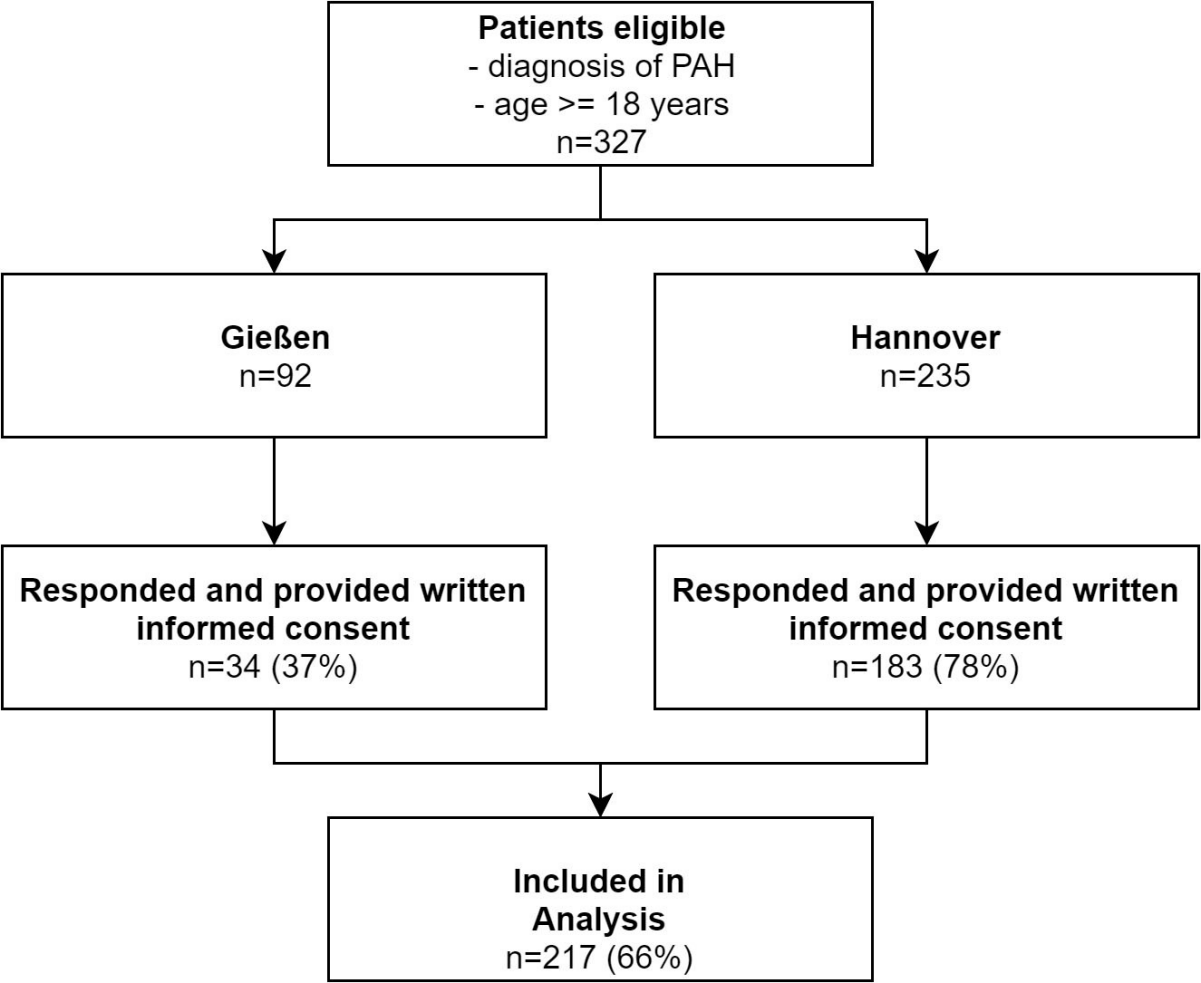
Flowchart of patient inclusion. PAH, pulmonary arterial hypertension.

Patients were contacted and interviewed between September 2019 and March 2020. A total of 327 patients were approached. Overall, 217 patients (66%) agreed to participate in the study, provided written informed consent, and took part in the interview (Figure 1). The patient characteristics are shown in Table 1.

**Table 1:** Characteristics of the patients at baseline.

	**All patients *n* = 217**	**Patients with depression or panic disorder*n* = 63 (29%)**	**Patients without depression or panic disorder *n* = 154 (71%)**	***p*-value**	**Effect size (Cohen's d^c^ or risk ratio^d^)**
Age (years)	56 (44–66)	49 (39–59)	59 (49–68)	**0.001**	0.549^c^
Female sex (%)	155 (71%)	46 (73%)	109 (71%)	0.741	1.03^d^
BMI (kg/m^2^)	26 (23–31)	26 (23–31)	25 (23–31)	0.569	−0.085^c^
**Diagnosis**					
IPAH, *n* (%)	122 (56%)	31 (49%)	91 (59%)	0.076	0.83^d^
HPAH, *n* (%)	25 (12%)	12 (19%)	12 (8%)	–	2.38^d^
Associated PAH, *n* (%)	70 (32%)	20 (32%)	50 (33%)	–	0.97^d^
Time since PAH diagnosis (years)	6 (3–11)	6 (2–11)	7 (3–12)	0.069	0.273^c^
**WHO FC**					
I/II, *n* (%)	112 (52%)	26 (41%)	86 (57%)	0.134	0.72^d^
III, *n* (%)	93 (43%)	35 (56%)	58 (38%)	–	1.47^d^
IV, *n* (%)	10 (5%)	2 (3%)	8 (4%)	–	0.75^d^
6MWD (m)	439 (353–521)	429 (344–525)	445 (359–523)	0.787	0.042^c^
NT-proBNP (ng/L), *N* = 62	184 (88–517)	199 (64–801)	181 (100–469)	0.455	−0.119^c^
DLCO (% pred.)	62 (47–74)	66 (57–75)	56 (42–73)	**0.021**	−0.426^c^
paO_2_, mmHg	67 (60–75)	68 (61–77)	66 (58–75)	0.072	−0.273^c^
**Hemodynamics at diagnosis**					
mPAP (mmHg)	48 (41–57)	48 (41–56)	49 (40–58)	0.720	0.066^c^
PAWP (mmHg)	9 (6–12)	8 (6–11)	9 (6–12)	0.231	0.189^c^
CI (L/min/m^2^)	2.4 (2.0–2.9)	2.4 (2.2–3.0)	2.4 (2.0–2.9)	0.512	0.103^c^
PVR (dyn·s·cm^−5^)	707 (501–947)	698 (510–958)	710 (500–930)	0.884	0.023^c^
**PAH medication**					
Monotherapy	44 (20%)	10 (14%)	34 (24%)	**0.037**	0.58^d^
Double combination therapy	102 (47%)	26 (41%)	76 (49%)	–	0.84^d^
Triple combination therapy	71 (33%)	27 (43%)	44 (29%)	–	1.48^d^
**Smoking status**					
Active, *n* (%)	24 (11%)	12 (19%)	12 (8%)	**0.027**	2.38^d^
Former, *n* (%)	31 (14%)	11 (18%)	20 (13%)		1.38^d^
Never, *n* (%)	162 (75%)	40 (64%)	122 (79%)		0.81^d^
Pack years	14 (5–25)	11 (5–19)	15 (5–26)	0.151	0.385^c^
**Sociodemographic items**					
Drinking (drinks per week)^a^	0.8 ± 2.0	0.9 ± 2.8	0.8 ± 1.5	0.600	−0.082^c^
Exercise score (points)	3 (2–4)	3 (2–3)	3 (2–4)	0.565^b^	0.107^c^
HADS-A (points)	6 (2–9)	10 (7–12)	4 (2–7)	**<0.001**	−1.46^c^
HADS-D (points)	5 (2–8)	9 (5–13)	4 (2–6)	**<0.001**	−1.344^c^
QoL-overall (points)	50 (38–75)	38 (25–63)	63 (50–75)	**<0.001**	0.686^c^
QoL-psych (points)	71 (58–79)	50 (41–67)	75 (63–83)	**<0.001**	0.853^c^
QoL-physical (points)	57 (45–75)	46 (32–64)	64 (50–75)	**<0.001**	0.818^c^

*Continuous variables are stated as median and interquartile ranges (IQR) and categorical variables are stated as n and percent (%), unless indicated otherwise*.

a *Mean and SD because of distribution of the data*.

b *Non-parametric Mann–Whitney U test because of ordinal scale of the variable*.

c *Cohen's d*.

d *Risk ratio*.

*BMI, body mass index; QoL, quality of life; HADS-A, Hospital Anxiety and Depression Scale—anxiety; HADS-D, Hospital Anxiety and Depression Scale—Depression; I/HPAH, idiopathic or heritable pulmonary arterial hypertension; WHO FC, World Health Organization Functional Class; 6MWD, 6-min walking distance; BNP, brain natriuretic peptide; NT-proBNP, N-terminal fragment of pro-brain natriuretic peptide; DLCO, diffusion capacity of the lung for carbon monoxide; paO_2_, mmHg, arterial pO_2_; mPAP, mean pulmonary arterial pressure; PAWP, pulmonary artery wedge pressure; CI, cardiac index; PVR, pulmonary vascular resistance; IQR, interquartile range. Statstically significant values as bold*.

Patients with mental disorders were younger, more likely to receive combination therapies for PAH, had a higher diffusion capacity of the lung for carbon monoxide, and were more likely to smoke than patients without mental disorders (Table 1). Disease severity as determined by FC, 6MWD, and NT-proBNP, hemodynamics at the time of PAH diagnosis, and alcohol consumption did not differ between the groups.

### Prevalence of Mental Disorders in PAH

**Figure 2:**
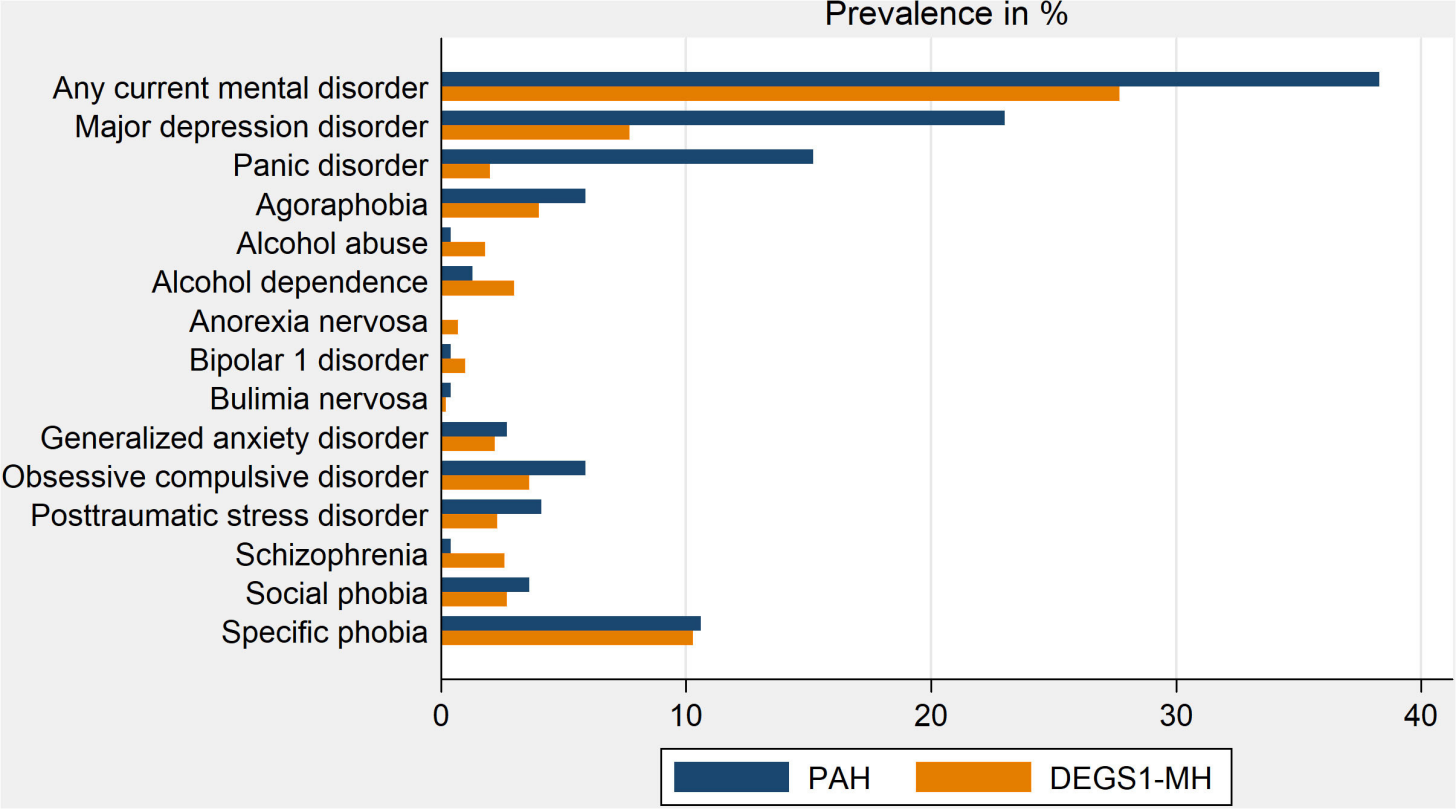
Comparison of mental disorders prevalence in patients with PAH and the German population. DEGS1-MH, Studie zur Gesundheit Erwachsener in Deutschland (German study of health status in adults)—Mental Health; PAH, pulmonary arterial hypertension.

In this cross-sectional study, current mental disorders were detected in more than one third of the patients, with major depressive disorder and panic disorder being the most prevalent mental illnesses (Table 2, Figure 2). Major depressive disorder and panic disorder were about 3 times and 8 times, respectively, more frequent in patients with PAH than in the general population. In contrast, the prevalence of other psychiatric illnesses was similar in patients with PAH and in the general population (Table 2, Figure 2).

**Table 2:** Prevalence of common mental disorders of PAH in comparison with data from the general German population (DEGS1-MH).

	**PAH n = 217**	**DEGS1-MHn = 5318**	**p-value**	**Risk ratio**
Any current mental disorder	38.2 (32.0–44.9)	27.7% (26.3–29.2)	**<0.001**	1.38
Alcohol abuse	0.4% (0.08–2)	1.8% (1.4–2.3)	–^a^	0.22
Alcohol dependence	1.3% (0.4–3)	3.0% (2.5–3.6)	–^a^	0.43
Schizophrenia	0.4% (0.08–2)	2.6% (2.1–3.2)	–^a^	0.15
Major depressive disorder	23% (17–29)	7.7% (6.9–8.6)	**<0.001**	2.99
Bipolar 1 disorder	0.4% (0.08–2)	1.0% (0.7–1.4)	–^a^	0.40
Panic disorder	15.2% (11–20)	2.0% (1.6–2.5)	**<0.001**	7.60
Agoraphobia	5.9% (3.5–9.9)	4.0% (3.4–4.7)	0.147	1.48
Social phobia	3.6% (1.8–7.1)	2.7% (2.2–3.4)	0.387	1.33
Generalized anxiety disorder	2.7% (1.2–5.8)	2.2% (1.8–2.8)	0.580	1.23
Specific phobia	10.6% (7.1–15.4)	10.3% (9.3–11.3)	0.889	1.03
PTSD	4.1% (2.2–7.7)	2.3% (1.8–2.8)	0.078	1.78
Obsessive compulsive disorder	5.9% (3.5–9.9)	3.6% (3.1–4.4)	0.066	1.64
Anorexia nervosa	0% (0–1.7)	0.7% (0.5–1.1)	–^a^	0.00
Bulimia nervosa	0.4% (0.1–2)	0.2% (0.1–0.3)	–^a^	2.00

*Psychological disorder prevalence rates per group stated as percent (%) and 95% CIs and risk ratios*.

a *Samples do not satisfy the standard binomial requirement*.

*PTSD, posttraumatic stress disorder; DEGS1-MH, Studie zur Gesundheit Erwachsener in Deutschland (German study of health status in adults)—Mental Health; PAH, pulmonary arterial hypertension. Statstically significant values as bold*.

Of note, 38.2% of PAH patients showed signs and symptoms of adjustment disorder after receiving the PAH diagnosis, and 53% of these patients suffered from at least one other psychiatric disorder at the time of the psychiatric assessment.

Male and female patients with PAH did not differ concerning prevalence rates of any mental disorder (data not shown).

### Diagnostic Value of the Hospital Anxiety and Depression Scale in Patients With PAH

**Figure 3:**
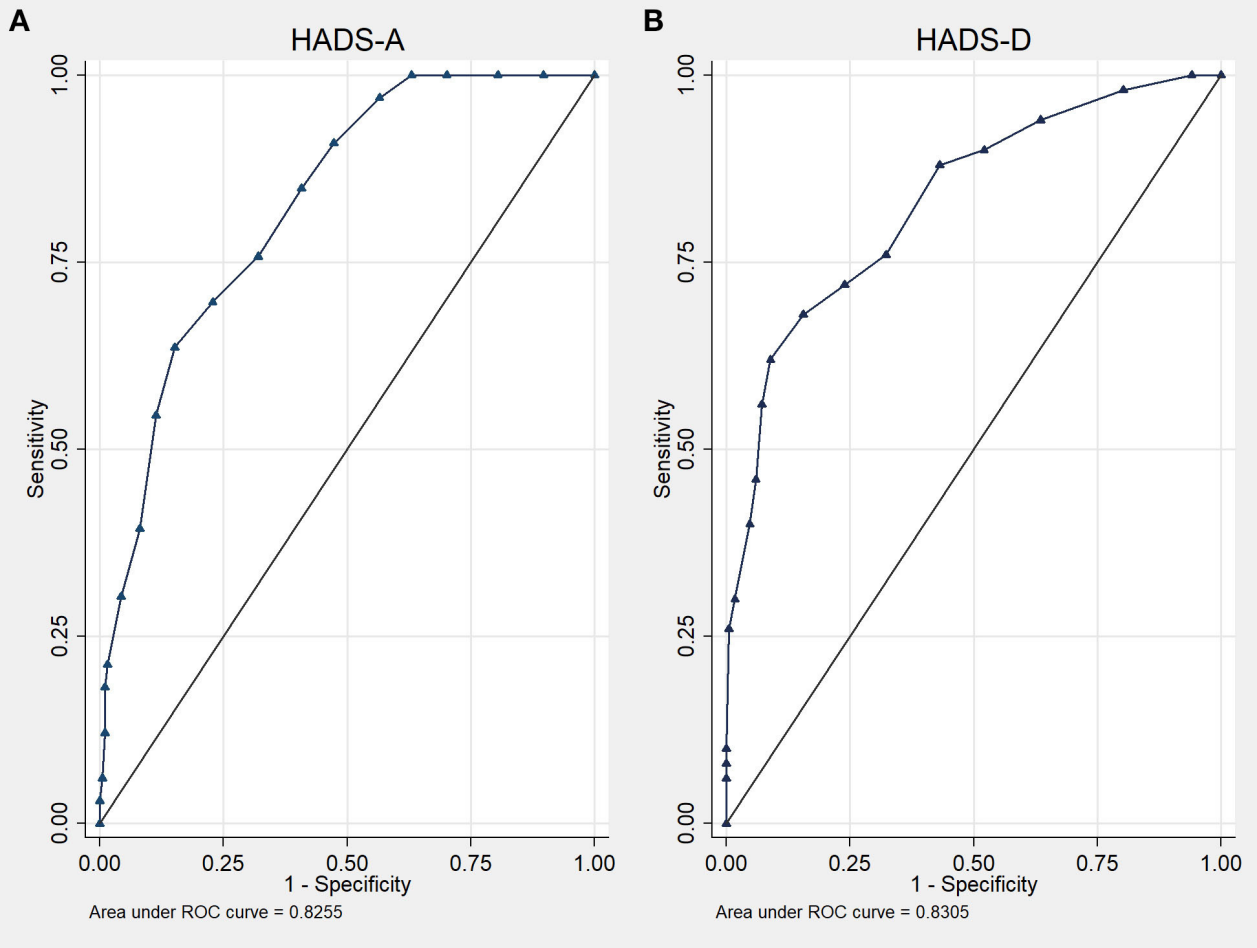
Receiver operating characteristic curves of the Hospital Anxiety and Depression Scale (HADS) for the detection of panic disorder **(A)** and depression disorder **(B)**.

At a cut-off value of ≥10 points, ROC curve analysis showed an AUC of 82.6% of the Hospital Anxiety and Depression Scale-A (95% CI 75.7–89.4%; *p* < 0.001) for the detection of panic disorder (Figure 3A). This resulted in a sensitivity of 63%, specificity of 85%, a positive predictive value of 42.9%, and a negative predictive value of 88.1%.

The AUC for the Hospital Anxiety and Depression Scale-D was 83.1% (95% CI 76.3–89.8%; *p* < 0.001) with an optimal cut-off of ≥9 points for the detection of major depressive disorder (Figure 3B). Sensitivity was 62%, specificity 80%, the positive predictive value was 47.7%, and the negative predictive value was 87.5%.

### Quality of Life in PAH

**Figure 4:**
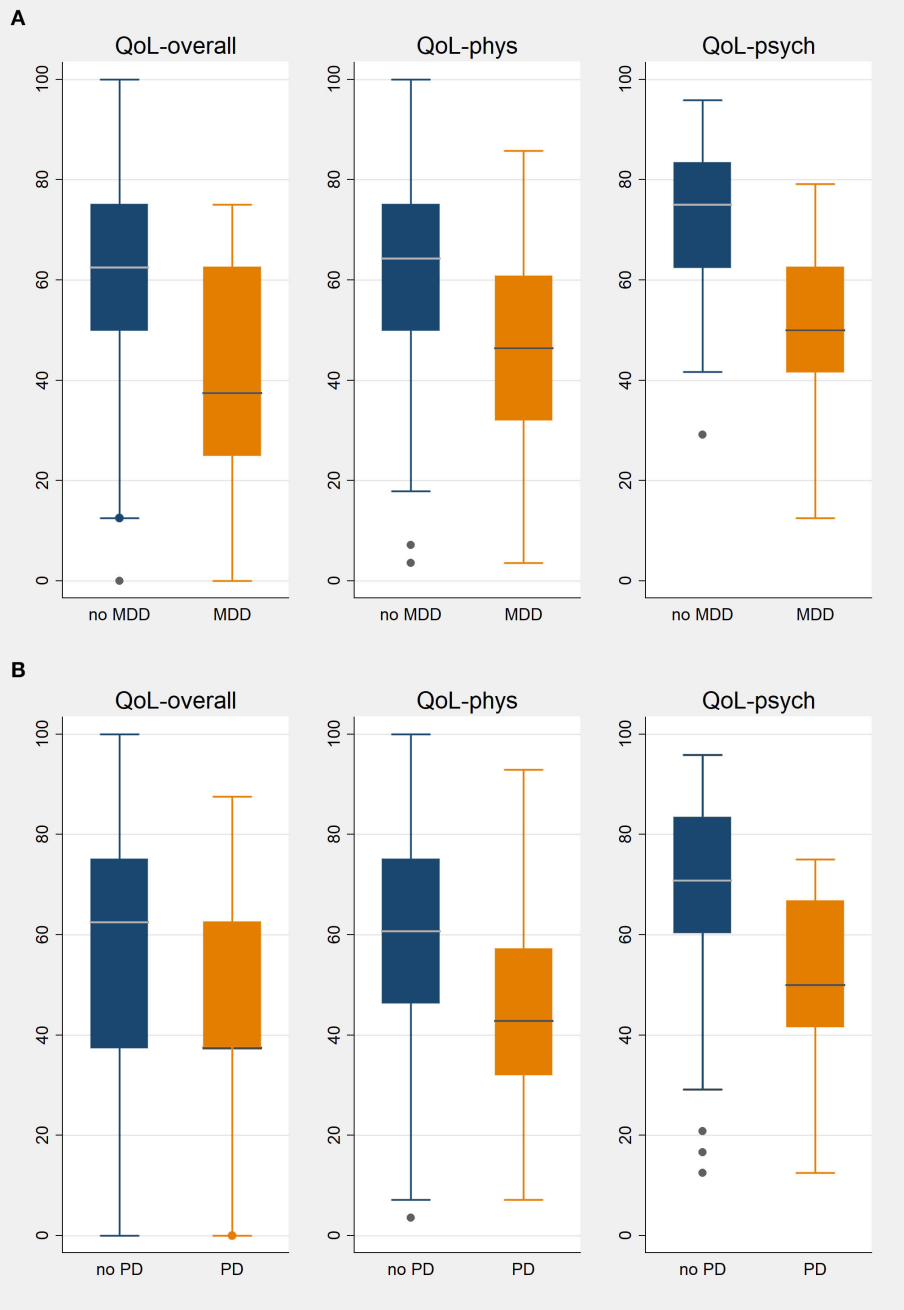
Quality of life in PAH patients with or without major depression disorder **(A)** or panic disorder **(B)**. QoL, quality of life; phys, physical; psych, psychological; MDD, major depression disorder, PD, panic disorder; box plots show medians and interquartile ranges.

The overall QoL of patients with PAH assessed by the WHOQOL-BREF tool was 50 (38–75) points. Patients with PAH and psychological disorders had a significantly lower QoL than those without psychological disorders: median, 38 (IQR, 25–63) points vs. median 63 (IQR, 50–75) points, *p* < 0.001. QoL for physical and psychological subscores were both significantly worse in patients with psychological disorders (Table 1, Figure 4). This was true notwithstanding the fact that the severity of PAH as determined by FC, 6MWD, and NT-proBNP at the time of the psychiatric assessment did not differ between patients with and without major mental disorders (Table 1).

### Determinants of QoL

By linear regression analysis, WHO FC, 6MWD, and mental disorders were significant determinants of overall and physical QoL. Each increase in WHO FC was associated with a 10-point decline in the overall QoL, and a decline in 6MWD by 10 m was accompanied by a 0.3-point decline in QoL. The presence of depression was associated with a 13-point decline in QoL and the presence of panic disorder was associated with an 8-point decline in QoL. Physical QoL decreased by 9 points per WHO FC worsening, by 14 points in the presence of depression, and by 8 points in the presence of panic disorder. Psychological QoL was determined mainly by the presence of depression disorder (−20 points) and panic disorder (−10 points), but also by 6MWD (0.4 points per 10 m).

## Discussion

To the best of our knowledge, this was the first study to systematically assess the prevalence of psychiatric disorders in patients with PAH by structured face-to-face interviews. The main findings can be summarized as follows: (1) more than one third of our patients had psychiatric disorders, mainly major depressive disorder and panic disorder, exceeding the prevalence of these mental disorders in the general population; (2) the presence of psychiatric disorders had a substantial impact on QoL; using linear regression analysis, major depression disorder was associated with a 13-point decline in the overall QoL on an ordinal scale ranging from 0 to 100 points, and the presence of panic disorder reduced QoL by 8 points; (3) more than one third of the patients under study reported signs and symptoms of adjustment disorder within the first 3 months after receiving the PAH diagnosis, and more than half of these patients developed other psychiatric disorders during the course of the disease; and (4) the Hospital Anxiety and Depression Scale may be a useful screening tool for the identification of probable cases of major depressive disorder and panic disorder in patients with PAH.

The high prevalence of psychiatric disorders in our cohort of patients with PAH was driven by depression and panic disorder, which were about 3 times and 8 times, respectively, more frequent than in the general population. This is in line with previous observations in patients with other chronic cardiovascular diseases such as congenital heart disease and heart failure (18, 19) and in other chronic diseases such as diabetes mellitus type 1 (20) or chronic obstructive pulmonary disease (21). In contrast, the prevalence of other major psychiatric disorders such as schizophrenia, bipolar disorders and alcohol dependence was not increased in our patients. Not unexpectedly, psychiatric disorders, foremost depression, had a major impact on QoL in our patients, contributing as much as the physical impairment to the reduced QoL. Hence, psychiatric disorders may become targets of therapy in this patient population.

To better understand and treat psychiatric disorders in patients with PAH, several key points have yet to be addressed. First, longitudinal assessment starting at the time of diagnosis is needed to distinguish between pre-morbid conditions and disorders that develop after the PAH diagnosis. Second, the role of adjustment disorders in the development of psychiatric disorders needs to be further clarified. In our study, 38.2% of the patients reported signs and symptoms associated with adjustment disorders after they received the PAH diagnosis. It is conceivable that this may have been the best time for therapeutic intervention, which may have prevented the development of manifest psychiatric disorders in some patients. Recently, successful psychological treatment of a patient with newly diagnosed PAH suffering from severe adjustment disorder using metacognitive treatment was described (22). Metacognitive treatment belongs to the wide spectrum of “third wave” cognitive behavioral therapies (23) and has the advantage of relatively short treatment duration. However, systematic studies and RCTs are not available at the moment, leading to the third key point, that the effects of psychotherapeutic interventions on psychiatric disorders and QoL in patients with PAH need to be studied in more detail.

Fourth and final, a proper diagnosis of psychiatric disorders in patients with PAH is a prerequisite for therapeutic interventions. Patients with PAH are mostly treated by cardiologists and pneumologists, i.e., physicians with limited experience in the detection of psychiatric disorders. As structured psychosomatic support is not yet part of the standard management of these patients in most pulmonary hypertension centers, a validated diagnostic tool might prove useful to identify or rule out the presence of psychiatric disorders. In the present study, we investigated the Hospital Anxiety and Depression Scale. This diagnostic tool was originally designed for medical inhospital patients and represents an established self-rating scale for non-psychiatric patients to detect depression and anxiety as a comorbidity (14). Several studies evaluated the diagnostic value of the HADS for various medical conditions. As a common feature, diagnostic accuracy varied according to the heterogeneity of patient groups and the underlying medical condition chosen, raising questions regarding its validity and concerning the optimal diagnostic cut-off points for particular physical diseases (24, 25). For example, in a recent study lower cut-off points for the detection of anxiety disorders and major depressive disorder were proposed for adults with congenital heart disease compared with the originally published cut-offs (19). In our study, the Hospital Anxiety and Depression Scale was found to have positive predictive values of 47.7 and 42.9%, respectively, and negative predictive values of 87.5 and 88.1%, respectively, for the detection of depression and panic disorder. Hence, the Hospital Anxiety and Depression Scale may be used as screening tool which may help non-experts to rule out the presence of depression and panic disorders, the most frequent psychiatric abnormalities in the present study. Pending further data, it might be advisable to recommend psychiatric or psychosomatic evaluation in patients with suspicious findings on the Hospital Depression and Anxiety Scale. However, it may be even more useful to develop specific questionnaires for patients with PAH, including tools with a high sensitivity for adjustment disorders, which can be used during the first 3 months after the diagnosis has been established.

Our study has strengths and limitations. The main strength is the inclusion of a relatively large set of patients with PAH who all underwent a structured face-to-face interview, which is considered the gold standard of psychiatric assessment. Limitations include the fact that only two centers from a single country participated. In addition, this cross-sectional study did not provide longitudinal data on the course of psychiatric disorders and also no data on the presence of psychiatric disorders before the development of PAH. In addition, the diagnosis of adjustment disorder after the PAH diagnosis was mainly based on retrospective assessments and may be confounded by recall bias.

In summary, our study showed a high prevalence of psychiatric disorders, especially major depressive disorder and panic disorder, in patients with PAH. The presence of these psychiatric disorders was associated with a profound reduction in QoL. In addition, our study found hints for a high prevalence of adjustment disorders after the diagnosis of PAH has been received. In some patients, adjustment disorders might be a starting point for the development of major mental disorders and may therefore provide an opportunity for early psychotherapeutic interventions. Further studies are needed to better understand the development of mental disorders in patients with PAH, to obtain reliable screening tools, and to assess therapeutic interventions.

## Data Availability Statement

The raw data supporting the conclusions of this article will be made available by the authors, without undue reservation.

## Ethics Statement

The studies involving human participants were reviewed and approved by Ethics committee, Hannover Medical School, Hannover, Germany. The patients/participants provided their written informed consent to participate in this study.

## Author Contributions

KMO was responsible for study design, implementation of the study, data collection, statistical analysis, data interpretation, and drafting the manuscript. TM was responsible for study design, implementation of the study, data collection, conducting the interviews, statistical analysis, data interpretation, and drafting the manuscript. JF was responsible for implementation of the study, data collection, statistical analysis, data interpretation, and drafting the manuscript. JCK and MJR were responsible for implementation of the study, data interpretation, and revising the manuscript. HG was responsible for study design, implementation of the study, data collection, data interpretation, and revising the manuscript. HAG was responsible for implementation of the study, data collection, data interpretation, and revising the manuscript. PF, RS, H-DK, IH, NL, and M-RD were responsible for study design, data interpretation, and revising the manuscript. MMH and KGK were responsible for study design, implementation of the study, statistical analysis, data interpretation, and drafting the manuscript. All authors contributed to the article and approved the submitted version.

## Conflict of Interest

KMO has received fees for lectures and/or consultations from Actelion, Janssen, MSD, Bayer, United Therapeutics, GSK, Janssen, Pfizer, and Acceleron, all outside the present study. HAG has received personal fees from Actelion, personal fees from AstraZeneca, personal fees from Bayer, personal fees from BMS, personal fees from GSK, personal fees from Janssen-Cilag, personal fees from Lilly, personal fees from MSD, personal fees from Novartis, personal fees from OMT, personal fees from Pfizer, and personal fees from United Therapeutics, outside the submitted work. HAG has received fees from Actelion, Bayer, Gilead, GSK, MSD, Pfizer, and United Therapeutics, outside the present work. MMH has received honoraria for lectures and/or consultations from Acceleron, Actelion, Bayer, GSK, Janssen, MSD, and Pfizer, all outside the present study. KGK has received honoraria for consultations and/or lectures from Eli Lilly, Janssen, Lundbeck, Neuraxpharm, Otsuka, Pfizer, Servier, Schwabe, Takeda, and Trommsdorff/Ferrer. The remaining authors declare that the research was conducted in the absence of any commercial or financial relationships that could be construed as a potential conflict of interest.
